# Supplementary material for: 3-NAntC: A Potent Crotoxin B-Derived Peptide against the Triple-Negative MDA-MB-231 Breast Cancer Cell Line
Source: Molecules. 2024 Apr 6;29(7):1646. doi: 10.3390/molecules29071646 (PMC11013444; doi:10.3390/molecules29071646)
Supplement: Supplementary file 1 [file molecules-29-01646-s001.zip › molecules-2922146-supplementary.pdf]

3-NAntC: A Potent Crotoxin B-Derived Peptide against the Triple-Negative MDA-MB-231 Breast Cancer Cell Line

Supplementary results.

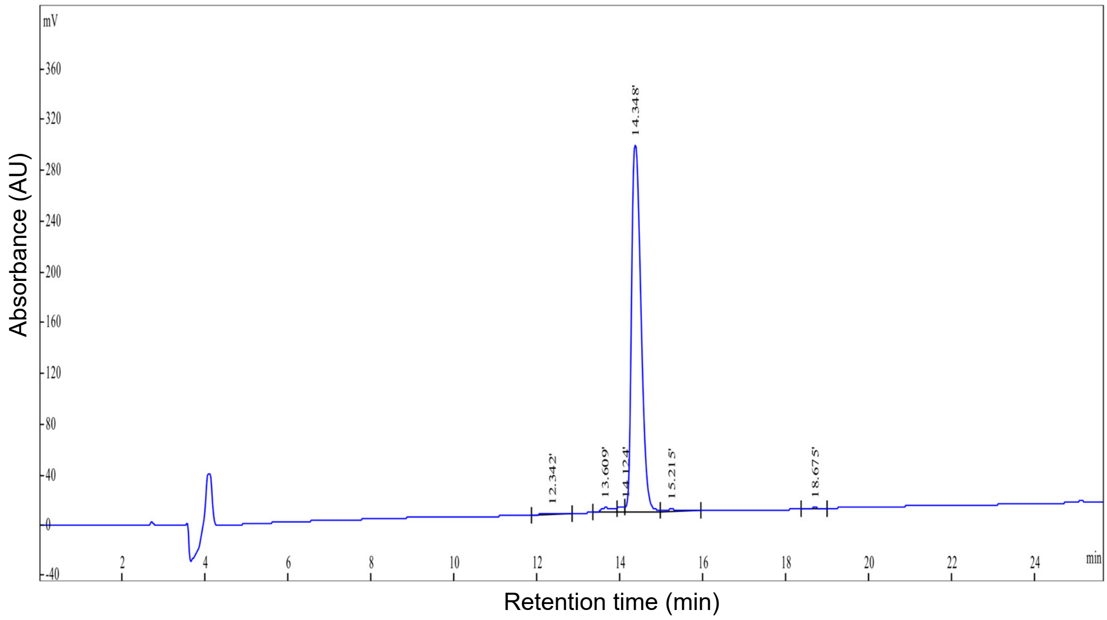

Peak No.	Ret Time	Height	Area	Conc.
1	12.342	1289	35239	0.7983
2	13.609	3409	71174	1.612
3	14.124	4266	44560	1.009
4	14.348	288295	4223309	95.67
5	15.215	1754	32466	0.7354
6	18.675	1101	7769	0.176
Total				100.0000

Supplementary Figure S1. **HPLC chromatogram of 3-NantC**. The peptide had a purity higher than 95%. Absorbance at the wavelength of 220 nm.

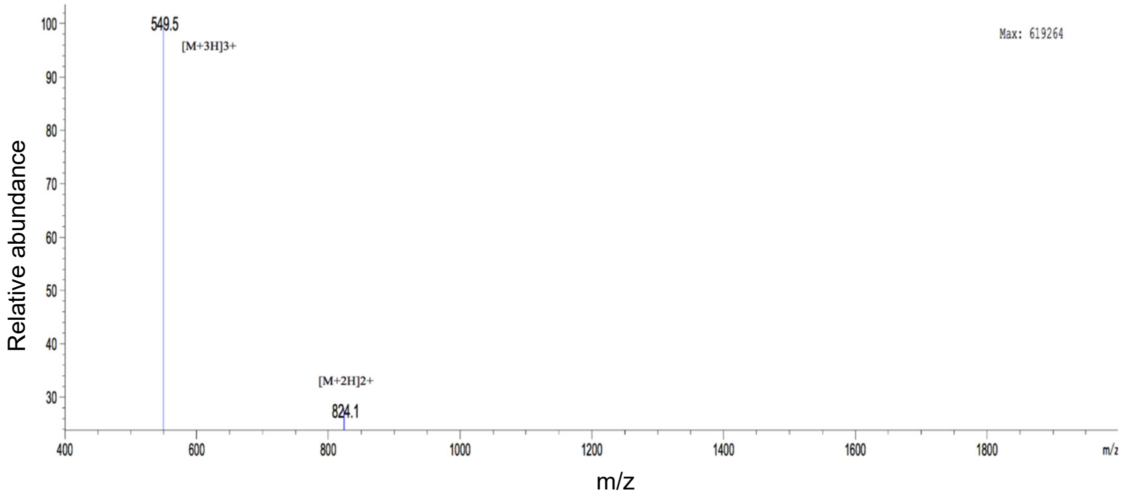

Supplementary Figure S2. **MS spectrum of 3-NAntC**. The mass spectrometry characterization with ESI ionization confirmed the peptide sequence [H]-MFYPDSRCRGPSET-[OH] (Molecular weight = 1,645.95 g/mol).

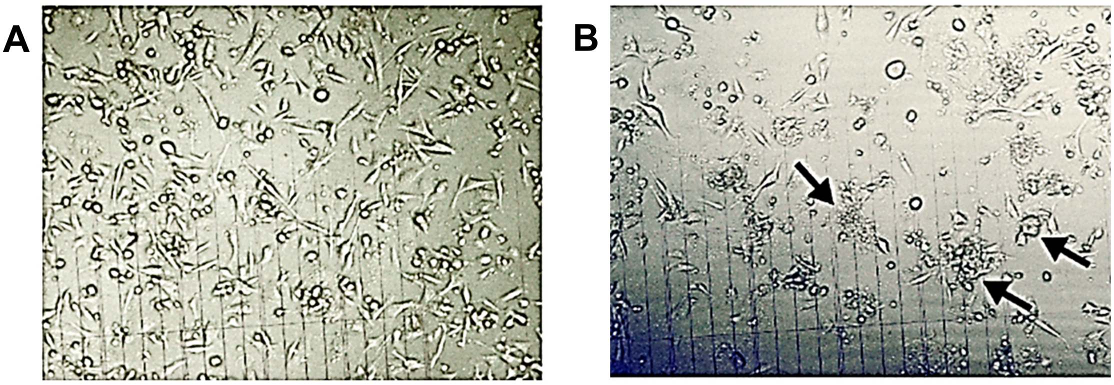

Supplementary Figure S3. **Antitumor effect of the peptide 3-NAntC against triple-negative breast cancer.** MDA-MB-231 cells were incubated in presence and absence of 3-NantC (1 µg/mL) for 24 hours. (A) Untreated triple negative tumor cells exhibiting a large number of cells. (B). Tumor cells after being treated with the peptide 3-NantC. The arrows indicate the tumor cells (MDA-MB-231), which are shown in smaller numbers and with vesicles compatible with apoptotic bodies when compared with the untreated tumor cells.

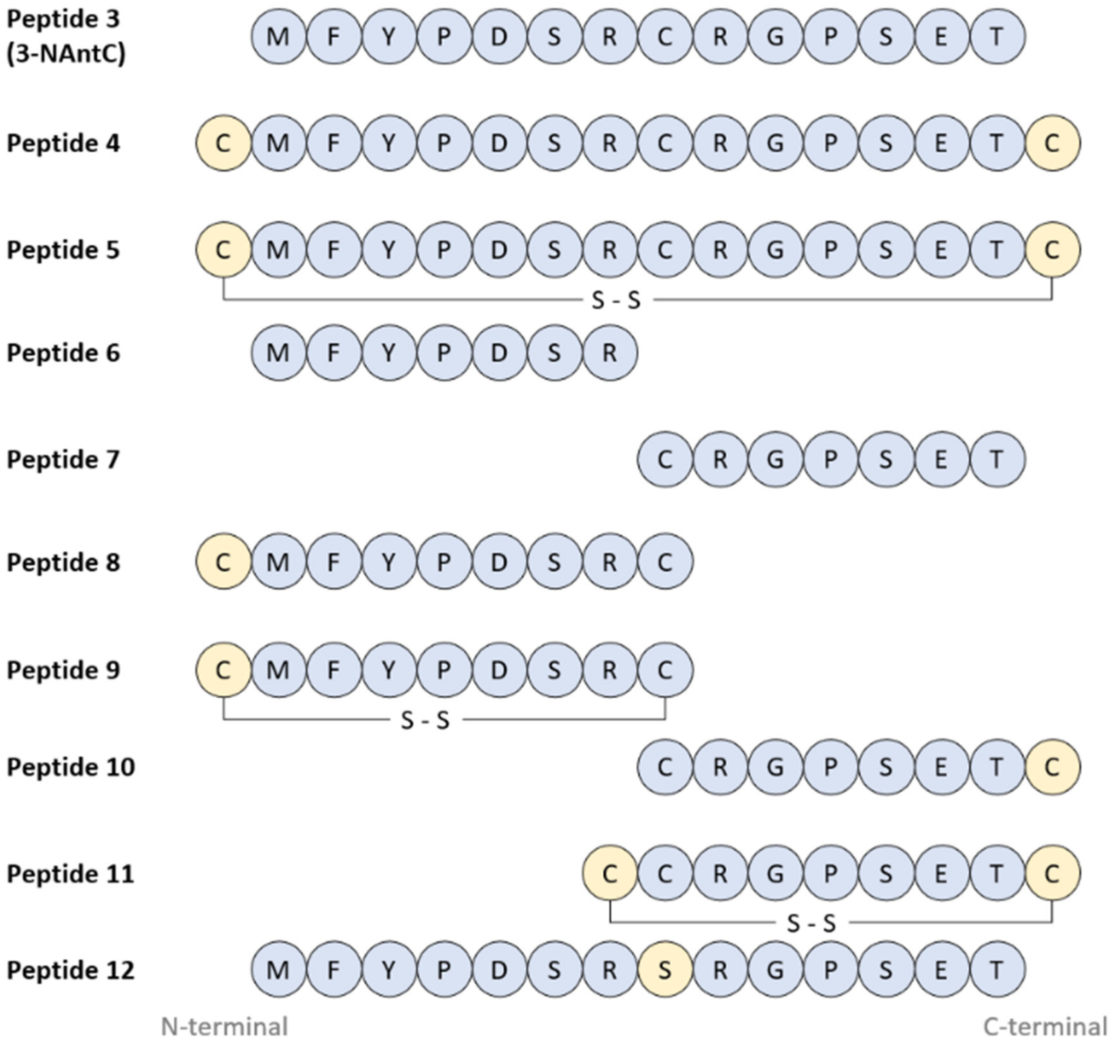

Supplementary Figure S4. Amino acid sequences of the 3-NAntC-derived peptides. The amino acids matching the original 3-NAntC sequence are shown in blue, while the additional amino acids are shown in light orange. Intramolecular disulfide bonds are denoted as ‘S-S’. The sequences are shown from the N- to the C-terminal regions.

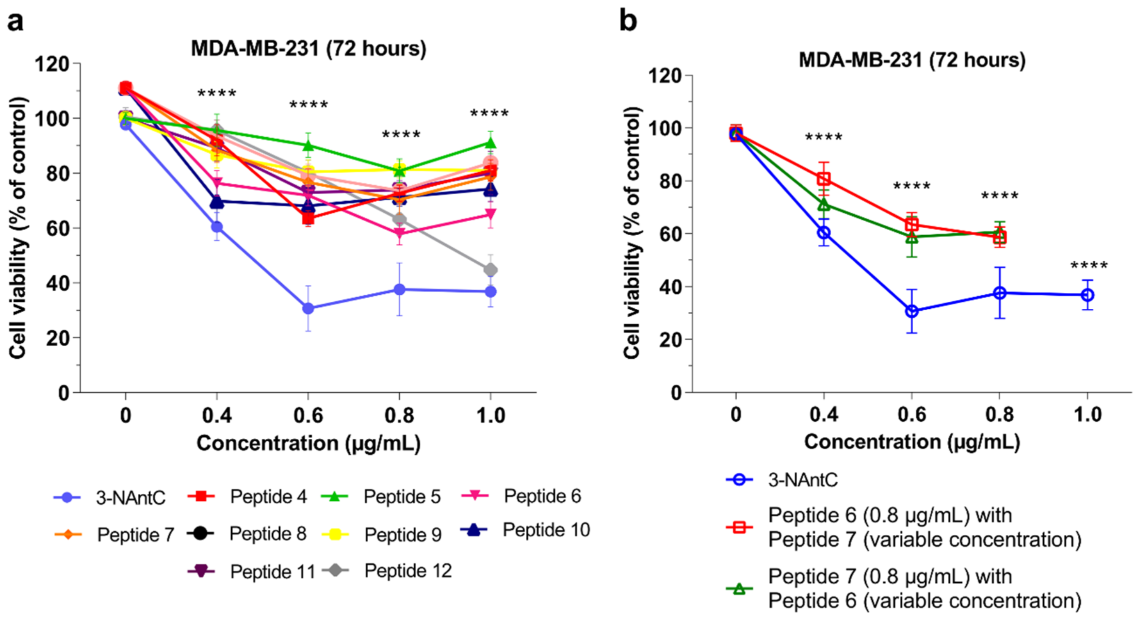

Supplementary Figure S5. Effect of 3-NAntC-derived peptides on TNBC cells (MDA-MB-231) viability. (A) MDA-MB-231 cells were treated with 3-NAntC or peptides 4 – 12 for 72 hours at the concentration range of 0.4 – 1.0 µg/mL. (B) Antitumor effect of 3-NAntC in comparison with the combination of the peptides 6 and 7. Cellular viability assay was conducted using the MTT method for the TNBC cell line. Data are shown as mean ± SEM of at least three independent assays in triplicate. Treatment groups for each peptide were compared to the respective negative control (PBS 0.02%) using the two-way ANOVA and the Bonferroni post hoc test. *$p < 0.05$, **$p < 0.01$, ***$p < 0.001$, and ****$p < 0.0001$ denote statistically significant differences for all the groups at a specific concentration.

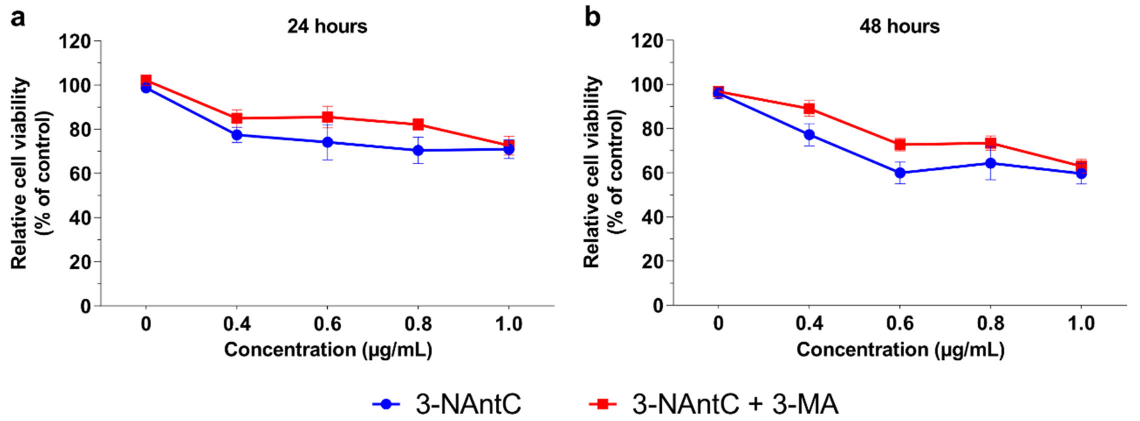

Supplementary Figure S6. **Effect of 3-methyladenine (3-MA) (an autophagy inhibitor) on the decrease of the MDA-MB-231 cell viability mediated by 3-NAntC.** MDA-MB-231 cells were treated with increasing concentrations of 3-NAntC in the presence or not of 3-methyladenine (3-MA) for 24 (A) or 48 hours (B). Cell viability was measured using the MTT assay. Data are shown as mean ± SEM of at least three independent assays in triplicate. Groups treated with 3-NAntC alone were compared to those treated with 3-NAntC + 3-MA (the autophagy inhibitor) using the one-way ANOVA and the Bonferroni posthoc test. No statistically significant differences were observed between the groups, $p > 0.05$.

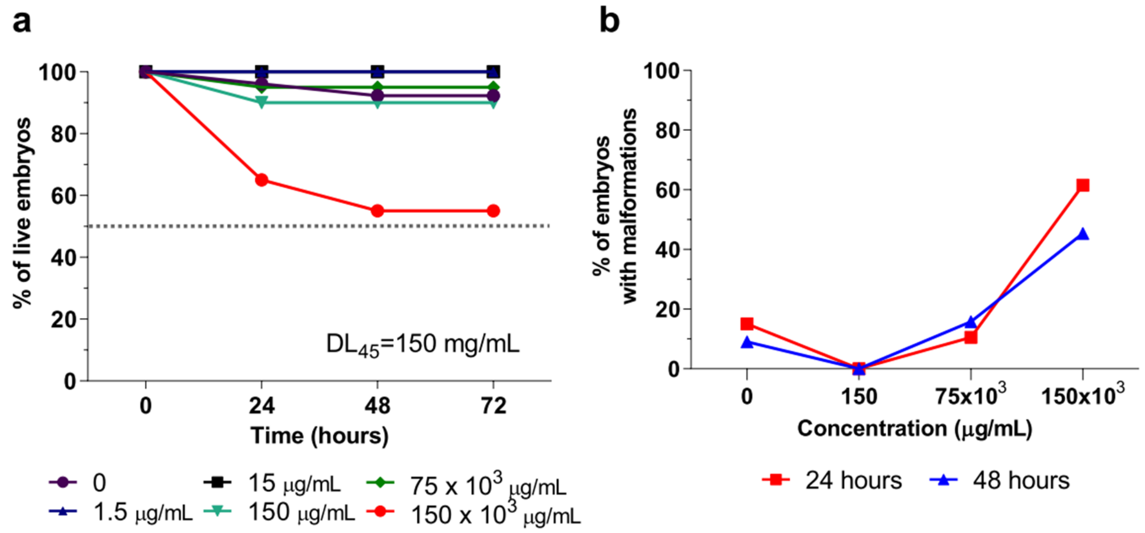

Supplementary Figure S7. 3-NAntC safety evaluation using zebrafish embryos. Embryo survival (A) and malformation in live embryos (B) were assessed following the exposure to 3-NAntC (1.5 – 150 x 10^3 μg/mL) for up to 72 hours. 3-NAntC exhibits low toxicity and malformation in zebrafish embryos up to 75 mg/mL. Data is shown as the percentage of live embryos and percentage of live embryos with malformation after the treatment with 3-NAntC (n=20 embryos/group).

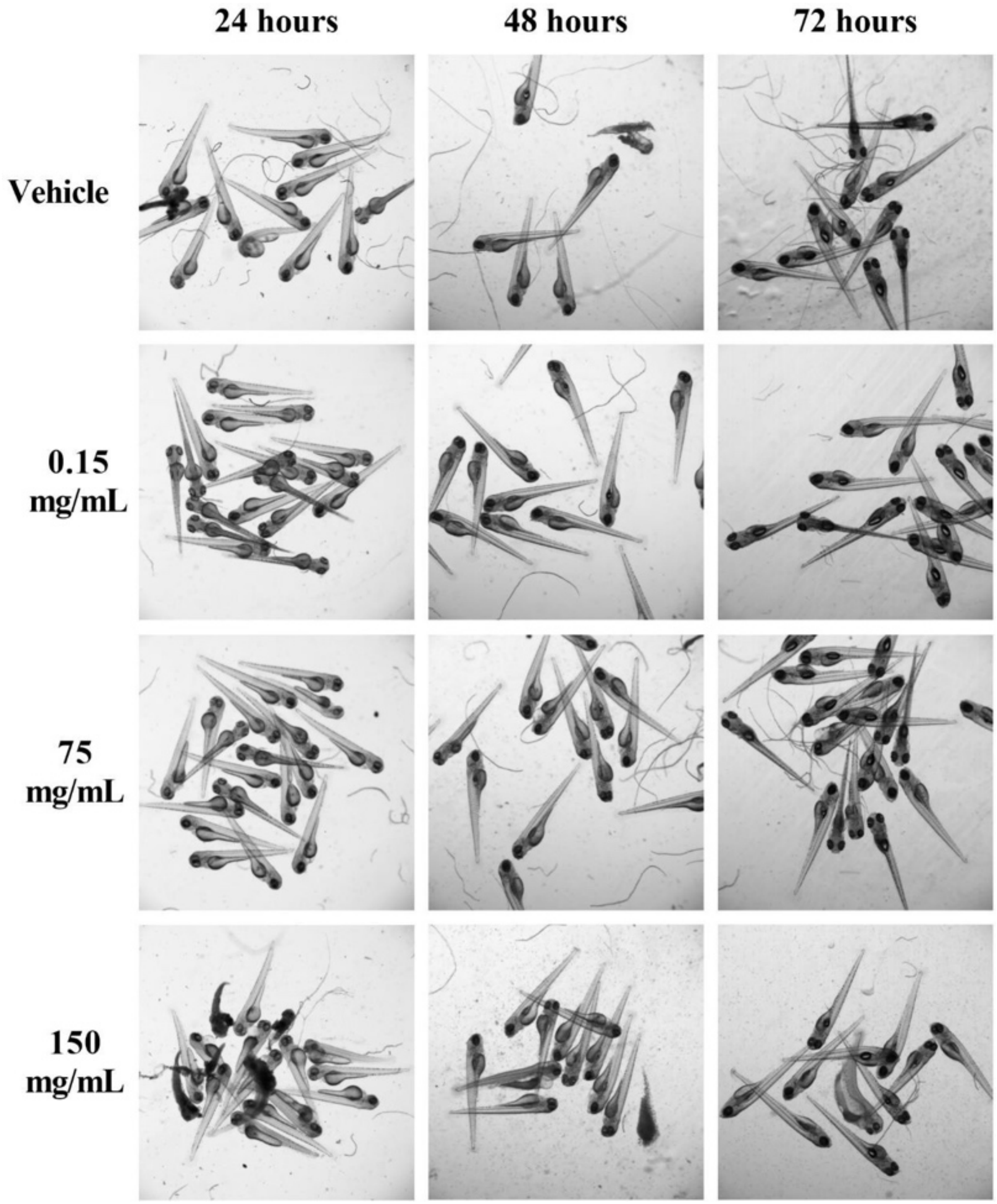

Supplementary Figure S8. Representative images of the tolerability test of 3-NAntC in zebrafish embryos. Zebrafish embryos were incubated with vehicle (control) or 3-NAntC peptide (0.15, 75, and 150 mg/mL) for 24, 48, or 72 hours.

Supplementary Table S1. Normality score for each data set used in the present study.

Data set			n	Shapiro-Wilk	
				score	p-value
Figure 2	24h	MCF10A Peptide 1	40	0.9527	0.7543
		MCF10A Peptide 2	51	0.8985	0.3217
		MCF10A Peptide 3	46	0.9157	0.4365
		MDA-MB-231 Peptide 1	29	0.9402	0.0514

		MDA-MB-231 Peptide 2	44	0.9541	0.0785
		MDA-MB-231 Peptide 3	37	0.9579	0.1737
Figure 2	48h	MCF10A Peptide 1	40	0.9278	0.5324
		MCF10A Peptide 2	57	0.9838	0.8265
		MCF10A Peptide 3	51	0.9609	0.6406
		MDA-MB-231 Peptide 1	21	0.9366	0.1865
		MDA-MB-231 Peptide 2	37	0.9589	0.1857
		MDA-MB-231 Peptide 3	39	0.9633	0.2291
Figure 3	24h	MCF10A	23	0.9283	0.1004
		HMEC	22	0.9638	0.1892
		HDFa	49	0.973	0.3173
		MDA-MB-231	58	0.9727	0.2155
		MCF7	28	0.9304	0.1633
Figure 3	48h	MCF10A	26	0.8954	0.1237
		HMEC	29	0.9451	0.1363
		HDFa	52	0.9566	0.5580
		MDA-MB-231	57	0.9821	0.5590
		MCF7	24	0.9409	0.1705
Figure 3	72h	MCF10A	28	0.9409	0.1164
		HMEC	37	0.951	0.1243
		HDFa	47	0.9619	0.1279
		MDA-MB-231	64	0.9382	0.0514
		MCF7	20	0.9724	0.8051
Figure 4	24h	MDA-MB-231 3-NAntC	60	0.9477	0.1210
		MDA-MB-231 Doxorubicin	68	0.9579	0.2192
		MDA-MB-231 Cisplatin	54	0.9635	0.2985
		HDFa 3-NAntC	50	0.9739	0.3317
		HDFa Doxorubicin	35	0.9243	0.1903
		HDFa Cisplatin	35	0.9532	0.2146
Figure 4	48h	MDA-MB-231 3-NAntC	52	0.9607	0.3843
		MDA-MB-231 Doxorubicin	66	0.9035	0.0858
		MDA-MB-231 Cisplatin	54	0.9795	0.4784
		HDFa 3-NAntC	54	0.9515	0.3790
		HDFa Doxorubicin	32	0.9286	0.3588

		HDFa Cisplatin	21	0.8565	0.05515
Figure 4	72h	MDA-MB-231 3-NAntC	83	0.8868	0.2450
		MDA-MB-231 Doxorubicin	69	0.8537	0.2010
		MDA-MB-231 Cisplatin	51	0.9732	0.3997
		HDFa 3-NAntC	53	0.9668	0.3470
		HDFa Doxorubicin	30	0.8243	0.1877
		HDFa Cisplatin	29	0.9127	0.3003
Figure 5	24h	MDA-MB-231	74	0.9587	0.3165
	48h	MDA-MB-231	74	0.9306	0.1608
		MDA-MB-231 G0/G1	19	0.8936	0.0574
		MDA-MB-231 S	23	0.9378	0.1649
		MDA-MB-231 G2/M	21	0.9696	0.4241
	72h	MDA-MB-231	67	0.9201	0.0585
		HDFa	35	0.9699	0.4405
Figure 6	24h	MDA-MB-231 Apoptosis	14	0.9511	0.5773
		MDA-MB-231 Necrosis	18	0.8226	0.0583
		MDA-MB-231 LDH	13	0.9251	0.2938
	48h	MDA-MB-231 Apoptosis	16	0.7916	0.2309
		MDA-MB-231 Necrosis	12	0.9499	0.6362
		MDA-MB-231 LDH	16	0.7745	0.2127
	72h	MDA-MB-231 Apoptosis	77	0.9294	0.3584
		MDA-MB-231 Necrosis	85	0.951	0.4746
		MDA-MB-231 LDH	72	0.9703	0.5867
